# Road safety strategies necessary in the second Decade of Road Safety

**DOI:** 10.7189/jogh.12.03081

**Published:** 2022-12-03

**Authors:** Becky PY Loo, Ka Ho Tsoi

**Affiliations:** 1 Department of Geography, The University of Hong Kong, Hong Kong SAR, China; 2 School of Geography and Environment, Jiangxi Normal University, China

While transport is integral to mobility and the economy, the hidden social costs of rapid motorisation are tremendous. As of 2019, there were around 1.3 million traffic fatalities, accounting for 2.3% of the total deaths and ranked 12th of the causes of mortality worldwide [1]. Road injuries also led to 79 million disability-adjusted life year (DALYs) in 2019, accounting for 3.1% of the global DALYs and ranked the 6th among other causes [1]. On average, road traffic deaths and injuries led to a 3% loss in Gross Domestic Product (GDP) [2]. Half of the global road traffic deaths are amongst the most vulnerable groups of pedestrians, cyclists, and motorcyclists [2]. Traffic fatality is the leading cause of death for young people aged 5-29 [2]. All these underline the equity dimension as these groups are bearing the costs of traffic deaths disproportionately. The serious negative transport externalities can dampen the goal of achieving social sustainability in transport [3]. Providing safe mobility for all aligns with the Sustainable Development Goals of “Sustainable Cities and Communities” and “Good Health” that “provide access to safe, affordable, accessible and sustainable transport systems” [4].

Despite a decline in global traffic fatality rates (per 100 000 population) in the past two decades from 19.1 to 16.7, road safety remains a prominent public health issue. First, there has been a general increase in the absolute number of global road traffic deaths since the 1990s, except during 2012 and 2015 [5]. Since then, it has rebounded and reached around 1.3 million [5]. It is expected that road traffic fatalities will rank among the top five causes of mortality by 2030 [6]. Second, there are still significant variations in traffic deaths rate (per capita). In 2019, the African average was around 1.6 times higher than the global average and 3.7 times higher than the European counterpart [5]. Also, traffic fatalities in low-income nations have been rising since 2013 and reached a record high of 28.34 per 100 000 population – a level 3 times higher than the high-income nations [5]. Third, the trend was far from the United Nations’ (first) Decade of Road Safety target of reducing road fatalities by 50%. With increased motorisation, the challenge of fatality reduction is huge. With the second Decade of Road Safety (2021-2030), one needs to go beyond modelling fatality records and measures, but to examine and advocate road safety strategies worldwide for effective policy formulation and implementation to “defatalise” transportation [3].

## A GLOBAL OVERVIEW

**Figure 1:**
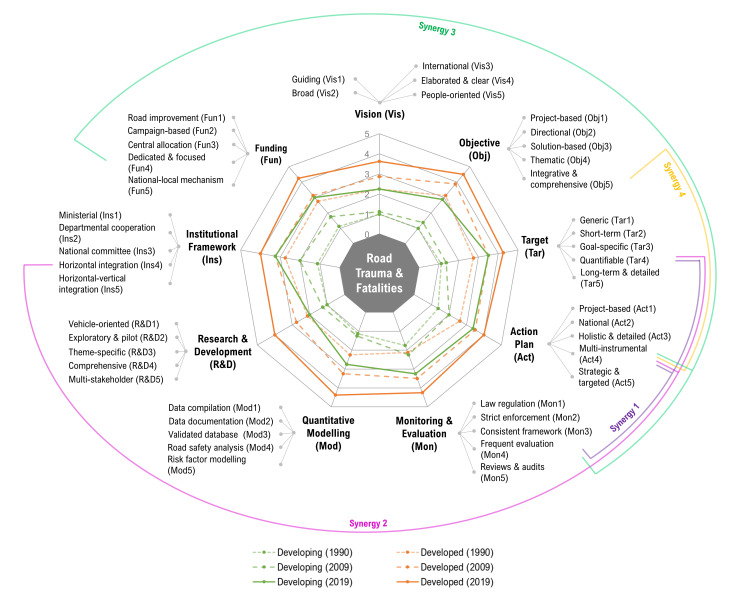
Road safety components and synergies for reducing road trauma and fatality.

To understand global road safety performances and desirable policy practices, we look at the situations in eight developed (Australia, Canada, France, Germany, Italy, Japan, United Kingdom, and United States) and eight developing nations (Brazil, China, India, Indonesia, Mexico, Nigeria, Russia and Turkey), which accounted for 75% of the global GDP and 60% of road fatalities [7]. Conceptually, transport decoupling is measured by the income elasticity of negative transport externalities, including traffic fatalities. Absolute strong decoupling, which indicates a concurrent reduction in traffic fatalities and growth of the economy, is the best outcome [3]. In road safety, the nine-component strategic framework is instrumental (elaborated below) [8]. **Figure 1** illustrates the nine core policy components. In each component, national policy performances are rated based on the levels of details, scope and degree of sophistication. The corresponding details of each rating are delineated next to the component. Finally, we adopt the method of Association Rule Mining (ARM) to identify underlying synergies among policy components in achieving transport defatalisation, which are visualised by different lines at the outer edge of the chart. Results are further elaborated below.

To look at the global divide, **Figure 1** summarises the mean scores of the nine road safety strategy components by developed and developing countries in 1990, 2009 and 2019. In general, both developed and developing nations have advanced their road safety policies over time. Under policy planning, the first component is “Vision” (Vis) and “Objectives” (Obj). Over the last three decades, the gap between developed and developing countries remained large. Among the developing nations, there was generally a lack of road safety vision and objectives before 2010.

**Figure:**
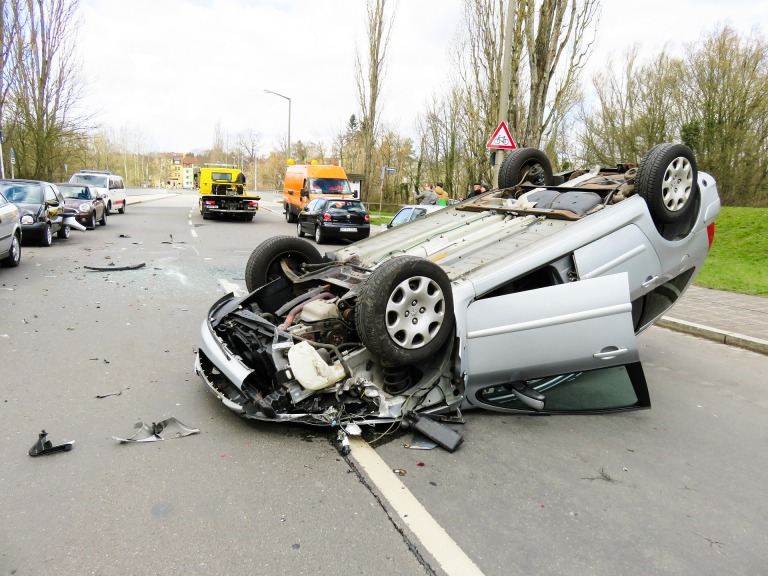
Photo: A car crash scene. Source: https://pixabay.com/photos/accident-automobile-damage-vehicle-1409012 (free to use under Pixabay Licence).

Another important feature of policy planning is “Targets” (Tar) and “Action Plan” (Act). Over the last three decades, the gap between the developed and developing countries has reduced substantially. Quantifiable targets were set under a 3 to 5-year timeframe. Some also pinpoint the reduction of risky driving behaviour and number of offenders. For action plans, both developed and developing countries have progressed significantly, too. Good practices include user-oriented measures targeting at vulnerable groups like motorcyclists, child passengers and pedestrians.

With “Monitoring and Evaluation” (Mon), the progress achieved in developing countries has been notable (**Figure 1**). Successful developing nations’ earlier tactics focused on deterring aggressive and impaired driving behaviour, such as speeding, drink-driving and not wearing seat belts. More recently, countries have proactively tackled new problems such as distracted driving and aggressive behaviour. Some countries conduct regular road safety reviews within a 2 to 3-year timeframe. The process provides feedback for adjusting the targets and associated policy instruments.

“Quantitative Modelling” (Mod) is an area that developing countries have been seriously lagging behind as a group (**Figure 1**). Brazil, China and Turkey have done better in this component with efforts to improve the official databases with spatial and temporal information on crashes, causes, and counts of injuries and fatalities. As “Research and Development” (R&D) is very much dependent on data and quantitative modelling, developing countries have lagged behind even more notably (**Figure 1**). Innovations in vehicle designs (eg, electronic stability control system & smart restraint systems) and regulatory techniques on roads (eg, US 5-star safety rating system) were practised in Japan, the UK and the USA.

As a group, developing countries have been catching up quickly in “Institutional Framework” (Ins) (**Figure 1**). In Brazil, China and Russia, a highest-level national council or committee was established. For vertical integration, Brazil, China and Mexico cooperated with international NGOs in initiating pilot plans and implementing safety campaigns based on the specific geographic context. In Australia, Canada, France and the UK, community groups work with local authorities to realise policy objectives at the local level. Overall, this indicates that the bridging of top-down and bottom-up approaches would be essential.

Generally, many countries have recognized the importance of “Funding” (Fun) but not all have a clear focus on targeted funding (**Figure 1**). Notably, the UK has offered financial incentives in local outreach programmes, driving training and infrastructure upgrades. Some developing nations (ie, China & India) have allocated funding for local authorities to enhance the capacity and responsiveness of rescue systems. The funding to improve post-crash management helps to reduce traffic fatalities.

## TOWARDS SYNERGY OF ROAD SAFETY COMPONENTS

With the launch of the Second Decade of Road Safety, the progress towards reducing road fatalities, especially in developing countries, must be accelerated. Based on the decoupling status and the evaluation scores of the nine policy components, synergies among road safety components can be identified by Association Rule Mining (ARM) using Artificial Intelligence techniques. To conduct ARM, the Apriori algorithm is applied to derive frequent item-sets from the database. This is determined by the minimum support rule, which indicates the number of items containing X to the total number of items. The minimum support rule is specified as 0.2 to capture common policy measures. Then, three additional association rules are established based on confidence, lift and conviction [9].

During the first Decade of Road Safety (2010-2019), four association rules are identified (**Figure 1**). The first is a three-item set with quantifiable targets within a realistic timeframe (Tar4), evaluation of road safety measures (Mon4), and a multi-instrumental action plan (Act4). The second but similar policy package is horizontal integration among different national department in road safety (Ins4), also combined with Tar4 and Act4. The third association rule consists of Mon4, Act4 and funding on specific road safety themes (Fun4). Finally, a two-item policy set comprises clear objectives aligning to multiple core strategies in a holistic framework (Obj5) and Act4.

To estimate the total fatality reduction associated with specific packages of policies identified by the association rules, the estimated total fatality reduction (EF) can be calculated by the average fatality rate reduction (per year) of nations implementing policy components in the respective rule (FR) multiplied by the total population of nation “i” in period “t”. The four association rules above can contribute to an annual reduction of 36 600 traffic deaths across the 16 countries.

## CONCLUSION

The first Decade of Road Safety has demonstrated the initial success and synergy of a multifaceted road safety strategy. It is encouraging to observe more developing nations adopting this approach. Nonetheless, the failure of an accountable leadership structure (capacity weakness) across international, country, provincial and city levels has remained a critical challenge. The overall accountability of a central agency needs to be strengthened by clear objectives that define core strategies in all sub-themes of road safety and direct actors to prioritise necessary actions. Official records of traffic injuries and fatalities have to be cross-validated. In large countries, the policy-implementation gap [10] between central and local authorities is notable. While political leadership is vital to effective road safety measures [11], holistic road safety strategies are urgently needed in the Second Decade of Road Safety.
